# Immunoglobulin G4-related sclerosing mastitis: an unexpected diagnosis for a breast mass

**DOI:** 10.1093/jscr/rjae378

**Published:** 2024-05-31

**Authors:** Loukia Koutsogeorgopoulou, Christos Damaskos, Nikolaos Garmpis, Eleni I Effraimidou, Iason Psilopatis, Anna Garmpi, Kleio Vrettou, Konstantinos Nikolettos, Afroditi Nonni

**Affiliations:** Department of Pathophysiology, Laiko General Hospital, Medical School, National and Kapodistrian University of Athens, Mikras Asias Street 16, 11527, Athens, Greece; Department of Emergency Surgery, Laiko General Hospital, Mikras Asias Street 16, 11527, Athens, Greece; N.S. Christeas Laboratory of Experimental Surgery and Surgical Research, Medical School, National and Kapodistrian University of Athens, Mikras Asias Street 16, 11527, Athens, Greece; N.S. Christeas Laboratory of Experimental Surgery and Surgical Research, Medical School, National and Kapodistrian University of Athens, Mikras Asias Street 16, 11527, Athens, Greece; Department of Surgery, Sotiria General Hospital, 152 Messogeion Ave, 11527 Athens, Greece; 1st Surgical Department, University Hospital of Alexandroupolis, Democritus University of Thrace, Dragana 68100, Alexandroupolis, Greece; Department of Obstetrics and Gynecology, University Erlangen Hospital, Universitaetsstrasse 21-23, Erlangen, Germany; First Department of Propedeutic Internal Medicine, Laiko General Hospital, Medical School, National and Kapodistrian University of Athens, Mikras Asias Street 16, 11527, Athens, Greece; Department of Cytopathology, Sismanogleio General Hospital, Sismanogleiou 1, 15126 Marousi, Athens, Greece; Department of Obstetrics and Gynecology, Democritus University of Thrace, Dragana 68100, Alexandroupolis, Greece; First Department of Pathology, Medical School, National and Kapodistrian University of Athens, Mikras Asias Street 75, 11527, Athens, Greece

**Keywords:** immunoglobulin, G4, IgG4, sclerosing, mastitis

## Abstract

Immunoglobulin G4-related disease is an immune-mediated condition comprised of a number of various disorders sharing unique pathologic, serologic, and clinical features. Diagnosis of immunoglobulin G4-related sclerosing mastitis is challenging as the clinical and imaging findings mimic breast malignancies or other types of inflammatory mastitis. Herein, we describe a case of a female patient with a painless palpable mass in her right breast. An excisional core biopsy led to the rare diagnosis of immunoglobulin G4-related sclerosing mastitis, and the patient received steroid treatment for a month. To date, the patient has remained disease-free without any recurrence. As immunoglobulin G4-related sclerosing mastitis is a very rare disease, further studies are needed to reach conclusions about the pathogenesis, diagnosis, and treatment of this entity.

## Introduction

Immunoglobulin G4-related disease (IgG4-RD) is an immune-mediated condition comprised of a number of various disorders sharing unique pathologic, serologic, and clinical features [1]. IgG4-RD can mimic a variety of different malignant, infectious, and inflammatory disorders, and it can affect one or multiple organs [2]. In the past, these diseases were regarded as isolated disorders whose pathophysiology was unknown [3, 4]. Over the past 20 years, a number of previously misdiagnosed cases have been newly diagnosed as IgG4-RD based on improved knowledge of this condition. The majority of IgG4-RD presents with tumor-like swelling of the affected organ, but diagnosis can be clinically difficult unless histopathologic findings include a lymphoplasmacytic infiltrate enriched in IgG4-positive plasma cells in the affected tissues [5]. Elevated serum concentrations of IgG4 are found in the majority of IgG4-RD patients [6, 7]. One of the IgG4-RDs is sclerosing mastitis, one of the rarest sub-forms of this group of diseases.

Herein, we describe the case of a female patient with a painless palpable mass in her right breast. Excisional core biopsy leads to the rare diagnosis of IgG4-related sclerosing mastitis (IgG4-RSM).

## Case presentation

A 40-year-old female presented with a painless mass and skin lesions in the right breast and concurrent skin lesions in her left arm. Her prior medical history was unremarkable. She had never smoked or consumed alcohol. She had never received hormonal therapy. This was the first time she felt a mass in her breasts.

Upon palpation, a mass could be felt in the upper medial quadrant of the right breast. The overlying skin was intact, and the form and size of the affected breast were approximately equal to those of the contralateral breast. The examination of the axilla was unremarkable. Complete blood count and basic biochemistry reports were unremarkable, apart from slightly elevated liver enzymes.

**Figure 1:**
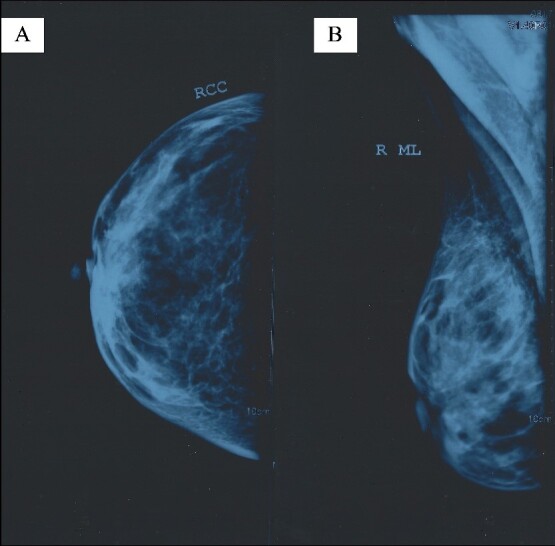
Right breast mammogram without pathological findings: (A) right craniocaudal (RCC) view and (B) right mediolateral (ML) view.

**Figure 2:**
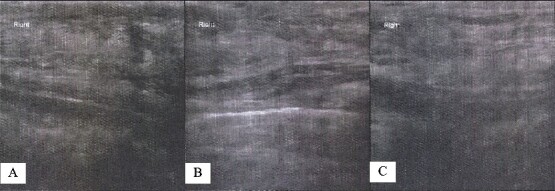
Right breast US findings. (A–C) Fibrocystic disease with cysts.

Mammograms were obtained, but they did not report any pathological findings (Fig. 1). Right breast ultrasound (US) reported fibrocystic disease and a cystic mass of 9.5 mm in diameter located at 11 o’clock, which was larger than the rest of the existing cysts (Fig. 2). Breast elastography reported oedema, diffuse thickening of the skin, and an increase in the vasculature of the upper medial quadrant of the right breast. No cystic or solid mass was reported. Additionally, an enlarged lymph node, 21 × 6 mm in size, was reported to be located in the right axilla. This lymph node had an echogenic medulla and an enlarged cortex.

**Figure 3:**
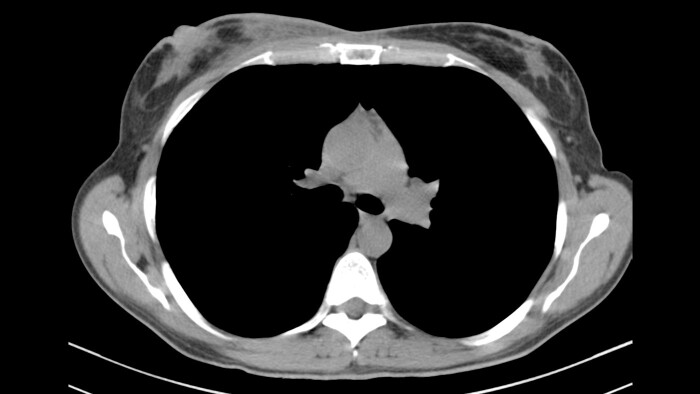
CT indicating heterogeneous enhancement and diffuse thickening of the skin of the right breast.

**Figure 4:**
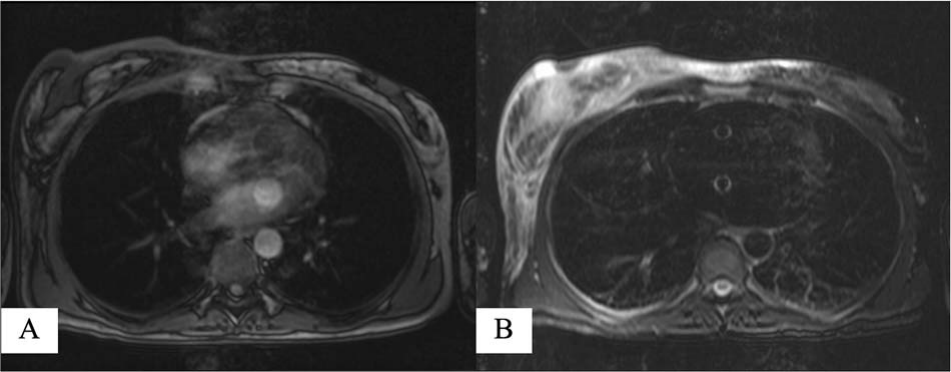
Magnetic resonance imaging reporting an area of 9.6 × 5 × 7.3 mm in the right breast enhanced by contrast uptake: (A) T1-weighted sequence and (B) T2-weighted sequence.

Due to the above findings, computed tomography (CT) (Fig. 3) and magnetic resonance imaging (MRI) were obtained. An MRI reported an area of 9.6 × 5 × 7.3 mm in the right breast enhanced by contrast uptake. The process infiltrated a segment of the overlying skin, and the MRI was labeled as Breast Imaging Reporting and Data System (BIRADS) category 4C (Fig. 4); therefore, a biopsy was requested.

**Figure 5:**
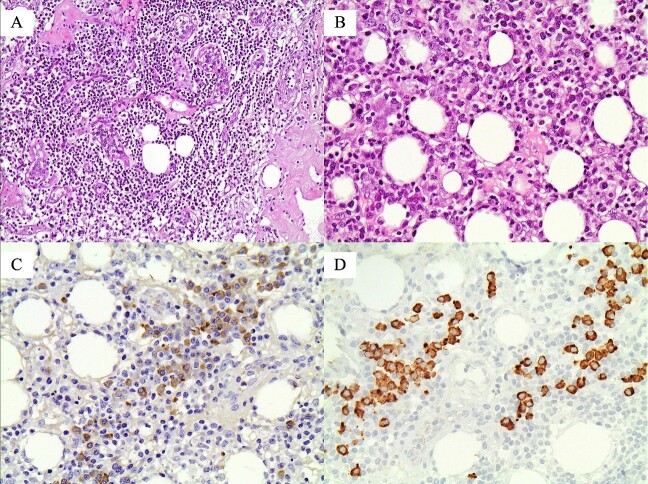
Histopathological findings, highly suggestive of IgG4-related sclerosing mastitis (IgG4-RSM): (A) Atrophic lobule with dense lymphoplasmacytic infiltration (original magnification ×200); (B) dense lymphoplasmacytic infiltration in the adipose tissue (original magnification ×400); (C) many IgG(+) plasmatocytes (original magnification ×400); and (D) more than 50 IgG4(+) plasmatocytes in this HPF (original magnification ×400).

As a fine needle aspiration (FNA) biopsy represents a rapid and low-cost alternative with high sensitivity and specificity to core biopsy [8], it was performed initially, and the findings were negative for malignancy. Because of the high suspicion of malignancy and the fact that core biopsy is associated with tumor cell seeding [9], an excisional biopsy was obtained. The result was negative for malignancy. Histopathology was positive for lymphoplasmacytic infiltrate enriched in IgG4-positive plasma cells, storiform fibrosis, prominent stromal sclerosis, and loss of breast lobules. Obliterative phlebitis was also present. An excision biopsy confirmed the diagnosis of IgG4-RSM. Actually, sections stained with hematoxylin and eosin (H + E) showed breast parenchyma with atrophic lobules and dense lymphoplasmacytic infiltration within or around them. The inflammatory cells were mainly T- and B-lymphocytes [CD3(+) and CD20(+), respectively], plasmacytes [CD18(+)], and fewer histiocytes. There was also stromal fibrosis, focally with a storiform pattern around the ducts, while the wall of some veins was invaded by the inflammatory cells, resulting in obliterative lesions. Immunohistochemically, there were many IgG(+) and IgG4(+) plasmacytes. Actually, there were 93 IgG4(+) plasmacytes per high power field (HPF) with the highest positive cells (>50), and the ratio of IgG4(+)/IgG(+) cells was 84% (>40%) (Fig. 5). The aforementioned histopathological findings were highly suggestive of IgG4-RSM. Serum IgG4 levels were 73 mg/dl.

**Figure 6:**
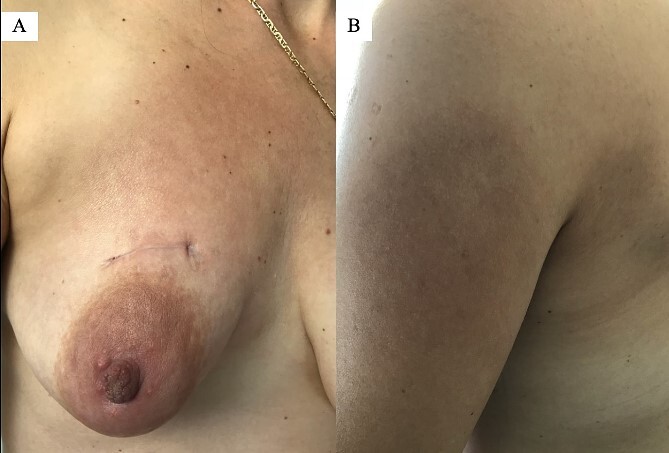
Since the mass excision, the skin lesions persisted: (A) right breast 20 days postoperatively and (B) left arm.

Twenty days after the mass excision, the skin lesions persisted (Fig. 6), so the patient was treated with steroids for the duration of a month. Five years after the diagnosis, she remains disease-free without any recurrence.

## Discussion

IgG4-RSM represents a rare member of the expanding IgG4-RD family. Diagnosis of IgG4-RSM is challenging as the clinical and imaging findings mimic breast malignancies or other types of inflammatory mastitis. Despite the lack of approved histopathological correlates specific to breast IgG4-RD, a biopsy is necessary for the diagnosis. As IgG4-RSM is a very rare disease, further studies are needed to understand the pathogenesis, diagnosis, and treatment of this entity.

## References

[1] Stone JH , Zen Y, Deshpande V. IgG4-related disease. N Engl J Med 2012;366:539–51. 22316447 10.1056/NEJMra1104650

[2] Kamisawa T , Zen Y, Pillai S, et al. IgG4-related disease. Lancet 2015;385:1460–71. 25481618 10.1016/S0140-6736(14)60720-0

[3] Kamisawa T , Funata N, Hayashi Y, et al. A new clinicopathological entity of IgG4-related autoimmune disease. J Gastroenterol 2003;38:982–4. 14614606 10.1007/s00535-003-1175-y

[4] Khosroshahi A , Stone JH. A clinical overview of IgG4-related systemic disease. Curr Opin Rheumatol 2011;23:57–66. 21124086 10.1097/BOR.0b013e3283418057

[5] Deshpande V , Zen Y, Chan JK, et al. Consensus statement on the pathology of IgG4-related disease. Mod Pathol 2012;25:1181–92. 22596100 10.1038/modpathol.2012.72

[6] Carruthers MN , Khosroshahi A, Augustin T, et al. The diagnostic utility of serum IgG4 concentrations in IgG4-related disease. Ann Rheum Dis 2015;74:14–8. 24651618 10.1136/annrheumdis-2013-204907

[7] Cheuk W , Chan JK. IgG4-related sclerosing disease: a critical appraisal of an evolving clinicopathologic entity. Adv Anat Pathol 2010;17:303–32. 20733352 10.1097/PAP.0b013e3181ee63ce

[8] Mitra S , Dey P. Fine-needle aspiration and core biopsy in the diagnosis of breast lesions: a comparison and review of the literature. Cytojournal 2016;13:18. 27651820 10.4103/1742-6413.189637 PMC5019018

[9] Maseki H , Jimbo K, Watase C, et al. Clinical significance of tumor cell seeding associated with needle biopsy in patients with breast cancer. Asian J Surg 2023;46:3700–4. 36732183 10.1016/j.asjsur.2023.01.026

